# Characterization of Botanical and Geographical Origin of Corsican “Spring” Honeys by Melissopalynological and Volatile Analysis

**DOI:** 10.3390/foods3010128

**Published:** 2014-01-27

**Authors:** Yin Yang, Marie-José Battesti, Jean Costa, Julien Paolini

**Affiliations:** Laboratory of Natural Product Chemistry, UMR CNRS 6134, Grimaldi Campus, Corsican University, BP 52, Corte 20250, France; E-Mails: yang@univ-corse.fr (Y.Y.); mjbattesti@univ-corse.fr (M.-J.B.); costa@univ-corse.fr (J.C.)

**Keywords:** honey, clementine and asphodel, melissopalynological analysis, HS-SPME, GC

## Abstract

Pollen spectrum, physicochemical parameters and volatile fraction of Corsican “spring” honeys were investigated with the aim of developing a multidisciplinary method for the qualification of honeys in which nectar resources are under-represented in the pollen spectrum. Forty-one Corsican “spring” honeys were certified by melissopalynological analysis using directory and biogeographical origin of 50 representative taxa. Two groups of honeys were distinguished according to the botanical origin of samples: “clementine” honeys characterized by the association of cultivated species from oriental plain and other “spring” honeys dominated by wild herbaceous taxa from the ruderal and/or maquis area. The main compounds of the “spring” honey volatile fraction were phenylacetaldehyde, benzaldehyde and methyl-benzene. The volatile composition of “clementine” honeys was also characterized by three lilac aldehyde isomers. Statistical analysis of melissopalynological, physicochemical and volatile data showed that the presence of *Citrus* pollen in “clementine” honeys was positively correlated with the amount of linalool derivatives and methyl anthranilate. Otherwise, the other “spring” honeys were characterized by complex nectariferous species associations and the content of phenylacetaldehyde and methyl syringate.

## 1. Introduction

The specificity of Corsican honeys is linked with the environmental characteristics of the island (biodiversity of flora, bioclimatic conditions and topography), the endemic black honeybee and typical hive management. Organoleptic and melissopalynological analysis have permitted Corsican honeys to be classified into six ranges: “spring”, “spring maquis”, “honeydew maquis”, “chestnut grove”, “summer maquis” and “autumn maquis”, according to the harvest season and the geographic location of the apiaries [1]. These honeys have been certified by two official designations of origin: the national Appellation d’Origine Contrôlée (AOC) and the European Protected Designation of Origin (PDO), both marketed as “Miel de Corse-Mele di Corsica” [2,3].

The organoleptic properties of the “spring” honey range are a light color (the lightest among the six ranges) associated with low-to-medium olfactory and aromatic intensities, sometimes with a slight acidity [1,2,3]. These honeys are described in terms such as floral, fresh fruit, or dry vegetal according to the vocabulary of odor and the aroma wheel [4]. Moreover, the physicochemical characteristics of “spring” honeys are low values of coloration and electrical conductivity. Finally, these honeys are harvested from April to May at low altitudes (below 400 m) on the coast, plains or valleys [1,2,3].

The Corsican “spring” honeys can be classified into two categories. First, honeys harvested in the oriental plain of the island. These cultivated zones are dominated by clementine orchards (*Citrus sinensis* × *reticulata*) associated with other *Citrus* species, *Actinidia sinensis* and various fruit trees. They are always surrounded by maquis; an evergreen scrub of vegetation from Mediterranean area. Second, honeys collected in ruderal and/or littoral maquis areas for their first flowering. Ruderal zones are characterized by herbaceous plants, especially *Asphodelus ramosus* subsp. *ramosus* (syn: *A. microcarpus* Salz et Viv.) associated with various species of *Fabaceae*, *Boraginaceae*, wild *Brassicaceae*, *Apiaceae* and *Asteraceae*. The coastal areas also showed a diversity of nectariferous and polleniferous resources [5].

Unifloral honeys from the *Citrus* genus, produced principally from oranges or lemons, are often found in the Mediterranean region (Italy, Spain, Greece, France and North Africa), but also in Israel, USA, Brazil and Mexico [6,7]. The nectar of *Asphodelus* species is frequently found in the composition of honeys from Mediterranean regions (Italy, Sicily, Corsica and Sardinia), but asphodel unifloral honey is produced mainly in Sardinia [8,9]. In Corsica, the *Asphodelus* genus is represented by three species: *A. ramosus* subsp. *ramosus*, *A. cerasiferus* and *A. fistulosus* [10]. *A. ramosus* subsp. *ramosus*, which flowers from March to May, was the more visited species.

The certification of geographical and botanical origins of Corsican honeys is conventionally based on the melissopalynological analysis of the entire pollen spectrum [5,11]. Furthermore, sensory characteristics and physicochemical parameters are also necessary to specify the botanical origin of honey [5,11,12]. However, this traditional approach is not precise enough to determine the predominant botanical origin exactly, especially when nectar resources are under-represented in the pollen spectrum. For this reason, the chemical composition of honeys has been used to complete the classical approaches of botanical origin determination. Thus, various extraction methods, such as headspace solid-phase microextraction (HS-SPME), simultaneous steam distillation-solvent extraction and ultrasound-assisted extraction associated with gas chromatography (GC) have been developed for the analysis of the volatile fraction of honeys [13]. Some volatile components, including methyl anthranilate, lilac aldehyde and *p*-menth-1-en-9-al, were therefore suggested as the chemical markers of citrus (species not specified) unifloral honey [13,14,15]. Moreover, Alissandrakis *et al*. [16] showed that the volatile fractions of citrus flowers (four species) and the corresponding honeys were dominated by linalool derivatives. The phenolic compound hesperetin was also proposed as a botanical indicator of Spanish citrus honeys for its high levels in nectar and honey [17]. Methyl syringate and/or phenylacetaldehyde were identified as characteristic components of nectar from *A. microcarpus* Salz et Viv. and corresponding unifloral honeys [18,19]. 

Several techniques (HS-SPME, infrared spectroscopy and ^1^H-nuclear magnetic resonance spectroscopy) have been used to distinguish Corsican and non-Corsican honeys, but these studies did not provide results for the differentiation of the botanical origin of different ranges of Corsican honey [20,21,22].

According to the geographical and botanical origins of Corsican “spring” honeys certified by melissopalynological analysis, the chemical composition of volatile fractions of honey samples was established using HS-SPME, GC and GC/mass spectrometry (MS). The aim of the study is to establish for the first time a multidisciplinary method for the qualification of Corsican “spring” honeys, based on relationships between the pollen spectrum, volatile chemical markers and some physicochemical parameters.

## 2. Experimental Section

### 2.1. Honey and Flower Sampling

In total, 41 Corsican “spring” honeys (samples 1–41) were selected from our reference bank of honey with AOC and PDO appellations. All these samples were directly packaged in a sealed pot and stored below 14 °C according to the optimal conditions of honey conservation indicated by Gonnet *et al.* [23]. The honey samples of three years of harvest (2004–2006) collected in April to June were provided from 12 Corsican producers. The apiaries were located from littoral to 400 m (principally under 100 m) in the oriental cultivated plain or in ruderal and/or maquis zone of thermo- and meso-Mediterranean levels. Clementine (*Citrus sinensis* × *reticulate*, six samples) and Asphodel (*Asphodelus ramosus* subsp. *ramosus*, six sample locations) flower specimens were collected in March–May 2009–2012. The nectar secretion during harvest period was ensured by the observation of foraging nectar by honeybees. Flowers samples were analyzed within 48 h.

### 2.2. Melissopalynological Analysis

In this study, melissopalynological analysis was performed using the method described by Yang *et al.* [24]. Identification of pollen in the “spring” honey was based on the comparison with laboratory’s own reference pollen-slides library and also carried out with the palynological expertise practice [5,11] developed for the characterization and the AOC and PDO control of Corsican honeys. Pollen analysis was allowed to establish a total pollen spectrum (qualitative analysis) and pollen density (quantitative analysis) for each honey sample. The identified taxa in the pollen spectrum were expressed in term of relative frequency (RF) and the pollen density was expressed as the absolute number of pollen grain in 10 g of honey (PG/10 g). 

### 2.3. Physicochemical Analysis

According to the description of Corsican honeys [1,5], two physicochemical parameters, coloration and electrical conductivity were chosen to complete the botanical origin characterization of Corsican “spring” honey. The honey coloration was measured using a Lovibond Comparator apparatus [25]. Results were expressed as millimeters (mm) Pfund. Electrical conductivity was measured at 20 °C with a conductivity meter micro CM2210 (CRISON, Spain) following the method described by Bogdanov [26] and expressed as milliSiemens per centimeter (mS/cm).

### 2.4. HS-SPME Extraction

Volatile fractions of honey and flower samples were extracted by HS-SPME with a divinylbenzene/carboxen/polydimethylsiloxane (DVB/CAR/PDMS, 30 μm) fiber (Supelco Sigma Aldrich). The optimization of HS-SPME parameters was performed using two honey samples (9 and 24) and two flower samples (clementine and asphodel flowers). These samples and subsequent analyses (all honey and flower samples studies) were performed in triplicate to ensure that the coefficient of variation (CV: ratio of standard deviation to the mean) of the major compounds and the sum of the total peak areas were always <15%. The samples analyzed were placed in a 20 mL vial. The parameter optimization was based on the sum of the total peak areas measured using a gas chromatography-flame ionization detection (GC-FID) system. For each sample (both honeys and flowers): the temperatures (25 °C, 50 °C and 70 °C), the equilibration times (30, 60 and 90 min) and the extraction times (15, 30 and 45 min) were tested in various experiments. The honey concentration in distilled water was optimized after six different experiments (0.5 g/mL, 1 g/mL, 1.5 g/mL and 2 g/mL) with Na_2_SO_4_ addition (1 g and 2 g). The maximum sum of the total peak areas was obtained from 4 g of honey sample with 4 mL of water and 2 g of Na_2_SO_4_ at a temperature of 70 °C, an equilibrium time of 90 min, and an extraction time of 30 min. The flower weight was optimized after three different experiments (1 g, 3 g and 5 g). For the Asphodel flowers, the maximum sum of the total peak areas was obtained from 3 g of sample at a temperature of 70 °C, an equilibrium time of 90 min, and an extraction time of 30 min. Otherwise, the best sampling conditions of Clementine flowers were 1 g of sample at room temperature (25 °C) with an extraction time of 15 min. Before sampling, the fiber was reconditioned for 5 min in the GC injection port at 280 °C. After sampling, the SPME fiber was consecutively inserted into the GC-FID and GC-MS injection ports for 5 min for desorption of volatile components, both techniques using the splitless injection mode. 

### 2.5. GC-FID and GC-MS Analysis

GC-FID analyses were performed using a PerkinElmer (Waltham, MA, USA) AutoSystem XL GC apparatus equipped with a FID system and a fused-silica capillary column (30 m × 0.25 mm, film thickness 1 μm) coated with Rtx-1 (PDMS). The oven temperature was programmed from 60 to 230 °C at 2 °C/min and then held isothermally at 230 °C for 35 min. The injector and detector temperatures were maintained at 280 °C. The samples were injected with an SPME inlet liner (0.75 mm i.d.; Supelco) using hydrogen as the carrier gas (1 mL/min). The retention indices of the compounds were determined relative to the retention times of a series of *n*-alkanes (C_5_–C_30_) with linear interpolation. The relative concentrations of components were calculated from the GC peak areas without using correction factors. Samples were also analyzed with a PerkinElmer TurboMass detector (quadrupole), coupled to a GC PerkinElmer AutoSystem XL, equipped with a fused-silica Rtx-1 capillary column. The ion source temperature was 150 °C, and the ionization energy was 70 eV. Electronic ionisation (EI) mass spectra were acquired over the mass range of 35–350 Da (scan time 1 s). Other GC conditions were the same as described for the GC-FID analysis. Identification of the components was based on: (1) the comparison of their GC retention indices (RI) on a nonpolar column, determined relative to the retention time of a series of *n*-alkanes with linear interpolation to the retention times of authentic compounds or data with the laboratory’s library; (2) the comparison of the RI and spectra with commercial mass spectra libraries [27,28].

### 2.6. Statistical Analysis

The statistical analysis of melissopalynological data was carrying out the methodology previously described by Battesti *et al.* [11]. In the case of “spring” honey, the inclusion of *Citrus* and *Asphodelus* pollen during the nectar foraging is low or very low because of pollen maturity or floral morphology. The “under-representation” of these pollen types and entire pollen spectrum were taken into account for the characterization and comparison of pollen spectrum from “spring” honeys. Principal component analysis (PCA) was carried out using the “PCA” function and canonical correspondence analysis (CCA) was performed with “CCA” function from R software (R Foundation—Institute for Statistics and Mathematics, Austria). CCA is a multidimensional exploratory statistical method in order to demonstrate the correlation between two sets of variables obtained from the same individual. 

## 3. Results and Discussion

### 3.1. Determination of Geographical and Botanical Origins of Corsican “Spring” Honeys

The analysis of 41 Corsican “spring” honeys allowed the determination of 92 taxa, including 64 nectariferous taxa and 28 only-polleniferous taxa (Table 1). A biogeographical analysis (biogeographical code: BC [5]) showed the diversity of biogeographical origins of these taxa. Mediterranean species (28 taxa, BC 1–3) associated with Eurasian and Atlantic species (13 taxa, BC 5–6) were well represented in the pollen spectrum. Additionally, cultivated species (four taxa, BC 99) were reported in more than 40% of honey samples. This distribution was consistent with the database of the characterization of the Corsican honey taxa directory [5,11].

To define the most representative taxa of Corsican “spring” honey, the presence ratio (PR) and the relative frequency (RF) distributions (mean, minimum, maximum, standard deviation and coefficient variation) of each taxon were reported. The pollen directory showed that 50 taxa (**T1**–**T50**) could be considered as regionally characteristic species of Corsican “spring” honey for their significant PR (>10%) and/or RF_max_ (>3%). This distribution of taxa was characterized by a wide diversity of nectariferous taxa in variable proportions associated with several only-polleniferous species. Among these taxa, two main only-polleniferous taxa, *Quercus* sp. **T1** (*Qeurcus* sp. (deciduous), *Q. ilex* and *Q. suber*) and *Cistus* sp. **T2** (*C. creticus*, *C. monspeliensis* and *C. salviifolius*), were present in all the samples analyzed, followed by *Castanea sativa* **T3** and *Fraxinus ornus* **T4** (PR > 90%). Additionally, we did not find a common predominant nectariferous taxon, unlike two previous studies [24,29]:“chestnut grove” honey predominated by *C. sativa* with PR = 100%, FR_max_ > 80% and FR_mean_ = 92.99% and “spring maquis” honey predominated by the “normal” pollen type of *Erica arborea* with PR = 100%, FR_max_ > 45% and FR_mean_ = 47.7%. Quite the contrary, this directly demonstrates a diversity of nectariferous taxa with various pollen representation types: for example, “over-represented” (**T7** and **T13**), “normal” (**T5**, **T6**, **T8**, **T9** and **T14**) and “under-represented” (**T17**, **T18** and **T22**) pollen types [5].

**Table 1:** Statistical analysis and biogeographical characteristics of Corsican “spring” honeys’ taxa.^*^

No ^a^	Type ^b^	Taxa	PR ^c^	Relative frequency (RF) ^d^					BC ^g^
				Mean	Min.	Max.	SD ^e^	CV ^f^	
**T1**	P	*Quercus* sp.	100	13.2	0.8	35.7	9.7	73.8	21-35-55-58
**T2**	P	*Cistus* sp.	100	8.5	0.3	33.3	6.3	74.4	21-29
**T3**	P	*Castanea sativa* ^h^	90	10.3	0.3	33.8	8.7	84.2	59
**T4**	P	*Fraxinus ornus*	90	3.3	0.3	22.3	5.0	153.2	58
**T5**	N, P	*Erica arborea*	85	7.8	0.2	35.5	8.7	112.3	21
**T6**	N, P	*Genista* form ^i^	83	6.0	0.3	31.5	8.0	134.8	14-21-29-51-62
**T7**	N, P	*Lotus* sp.	76	5.3	0.3	52.8	9.5	178.3	21-51
**T8**	N, P	*Salix* sp.	73	6.3	0.2	29.9	7.4	117.1	51-52
**T9**	N, P	*Trifolium* sp.	71	14.2	0.4	53.5	16.8	117.9	21-31-51
**T10**	N, P	*Rubus* sp.	71	3.6	0.4	11.7	3.4	94.6	31-35
**T11**	N, P	*Prunus* form ^j^	66	3.0	0.2	24.1	4.7	155.6	99-54
**T12**	P	*Eucalyptus* sp.	63	2.1	0.3	15.5	3.1	148.4	99
**T13**	N, P	*Echium* sp.	59	10.5	0.6	71.1	15.6	148.2	31
**T14**	N, P	*Apiaceae*	59	4.0	0.2	17.5	4.4	109.6	nd
**T15**	P	*Actinidia sinensis*	49	4.2	0.3	16.1	4.5	107.7	99
**T16**	N, P	*Brassicaceae* others	49	2.8	0.3	14.7	3.3	118.6	nd
**T17**	N, P	*Lavandula stoechas*	49	1.8	0.4	10.1	2.2	124.1	21
**T18**	N, P	*Citrus* sp.	44	6.1	0.2	16.1	5.2	86.6	99
**T19**	N, P	*Vicia* form	44	3.0	0.3	11.8	3.2	107.1	nd
**T20**	P	*Pistacia lentiscus*	44	3.0	0.5	9.3	2.6	88.0	29
**T21**	N, P	*Asteraceae Galactites* form	44	1.9	0.2	5.2	1.7	93.6	21
**T22**	N, P	*Asphodelus ramosus* subsp. *ramosus*	44	0.7	0.2	2.9	0.7	96.5	21
**T23**	P	*Scrophulariaceae* others	39	0.9	0.3	4.5	1.0	114.4	nd
**T24**	P	*Phillyrea* sp.	37	3.0	0.3	13.3	3.8	125.5	25
**T25**	P	*Olea* sp.	37	1.0	0.4	3.6	0.8	74.3	21
**T26**	N, P	*Viburnum tinus*	34	1.9	0.3	16.2	4.2	225.9	21
**T27**	N, P	*Asteraceae* (fenestrated type)	29	1.1	0.3	3.2	1.0	94.0	21-94
**T28**	N, P	*Rosa* sp.	27	1.2	0.3	4.5	1.3	108.1	31-51
**T29**	P	*Myrtus communis*	24	0.9	0.3	1.6	0.5	52.0	21
**T30**	N, P	*Fabaceae* others/*Dorycnopis* form	24	0.6	0.3	1.4	0.3	54.5	nd
**T31**	P	*Plantago* sp.	24	0.5	0.3	0.9	0.2	33.8	nd
**T32**	N, P	*Asteraceae Achillea* form	22	0.8	0.2	2.6	0.7	94.0	21-94
**T33**	P	*Poaceae*	22	0.6	0.2	1.2	0.3	54.0	nd
**T34**	N, P	*Crataegus monogyna*	20	2.1	0.3	7.9	2.7	130.8	51
**T35**	N, P	*Jasione montana*	17	2.0	0.3	10.0	3.5	176.0	54
**T36**	N, P	*Rosaceae* others	17	1.2	0.3	3.0	1.0	85.6	nd
**T37**	N, P	*Asteraceae Dittrichia* form	17	1.0	0.2	1.9	0.8	74.4	21-94
**T38**	N, P	*Rhamnus* sp.	15	1.0	0.3	3.3	1.1	117.6	21
**T39**	N, P	*Psoralea bituminosa*	15	0.7	0.3	1.6	0.5	75.6	31
**T40**	N, P	*Knautia* sp.	15	0.5	0.3	0.9	0.3	52.8	31
**T41**	N, P	*Lupinus angustifolius*	12	4.8	0.3	18.9	8.0	166.1	21
**T42**	P	*Cytinus hypocistis*	12	0.9	0.4	1.8	0.7	77.6	29
**T43**	N, P	*Hedera helix*	12	0.8	0.3	1.3	0.4	46.1	65
**T44**	N, P	*Liliaceae others*	12	0.4	0.3	0.6	0.2	45.8	nd
**T45**	N, P	*Allium* sp.	12	0.4	0.3	0.6	0.2	42.9	21-25
**T46**	N, P	*Acacia dealbata*	12	0.4	0.3	0.4	0	13.3	99
**T47**	P	*Alnus* sp.	7	2.3	0.3	6.1	3.3	146.5	51
**T48**	N, P	*Dorycnium* sp.	10	1.5	0.3	3.2	1.2	83.3	35
**T49**	N, P	*Rosmarinus officinalis*	10	1.7	0.4	3.0	1.3	77.9	21
**T50**	P	*Vitis vinifera*	7	1.5	0.4	3.0	1.3	89.4	99

^a^ Order of taxa were classified by decreasing presence ratio (PR). ^b^ Type of taxa: P, polleniferous taxa; N, nectariferous taxa [5]. ^c^ PR: presence ratio, number of honey samples presented/41 samples, expressed as %. ^d^ Mean, Min., Max. values expressed as relative frequency RF (number of specify pollen counted/total pollen counted). ^e^ SD: standard deviation. ^f^ CV: coefficient variation. ^g^ Biogeographical Code, according to Battesti [5]: 1—*Endemic*: 14 Mediterraneo-montane origin; 2—*Steno-Mediterranean*: 21 Wider stenomedit., 25 Western stenomedit., 29 Western macaronesian stenomedit.; 3—*Eury-Mediterranean*: 31 Wider eurymedit., 35 Western eurymedit.; 5—*Eurasian*: 51 Wider eurasian, 52 Eurasian, 54 European-caucasian, 55 European, 58 South east european, 59 Southern European; 6—*Atlantic*: 62 Subatlantic, 65 Atlantic Mediterranean; 94 sub-Cosmopolitan; 99 Cultivated plants; nd: not defined. ^h^ Castanea sativa, taxa of “over-represented” type, could be considered as only-polleniferous taxon according to its RF (<40%) and lower pollen density taking into account its over-represented pollen type [6,24]. ^i^ Genista form contained essentially *Genista corsica*, and also *Cytisus villosus*, *Calicotome spinosa* and *Calicotome villosa*. ^j^ *Prunus* form contained *Prunus* sp. and other fruit tree. * Forty two other determined taxa (PR < 10%): *Populus* sp., *Boraginaceae* others, *Rumex* sp., *Ostrya carpinifolia*, *Ilex aquifolium*, *Platanus* sp., *Silene gallica*, *Stachys glutinosa*, *Anthyllis hermanniae*, *Papaver* sp., *Urticaceae*, *Reseda* sp., *Aesculus hippocastanum*, *Carpobrotus* sp., *Cercis siliquastrum*, *Potentilla* form, *Ranunculaceae*, *Corylus avellana*, *Asteraceae Helichrysum* form, *Arbutus unedo*, *Erica* others, *Cupressaceae*, *Sambucus ebulus*, *Anemone hortensis*, *Smilax aspera*, *Cynoglossum creticum* form, *Amaryllidaceae*, *Cyperaceae*, *Helleborus lividus* subsp. *corsicus*, *Mercurialis annua*, *Robinia pseudoacacia*, *Clematis* sp., *Chenopodiaceae*, *Caryophyllaceae* others, *Borago officinalis*, *Centaurea* sp., *Verbascum* sp., *Teucrium* sp., *Centaurium erythrae*, *Veronica* sp., *Asteraceae* others, *Buxus sempervirens* (according to decreasing PR).

According to these considerations, two groups of honeys could therefore be distinguished, based not by their FR distributions, but by characteristic associations of taxa (Table 2, Table S1-supplementary materials). The first group included 18 samples (group I: 1–18) and was characterized by the association of cultivated taxa: *Citrus sp.* **T18** and *A. sinensis* **T15** (PR 100% in 18 samples) followed by *Prunus* form **T11** and *Olea* sp. **T25**. *Citrus* sp. contained essentially *C. sinensis* × *reticulata*, which possessed an under-represented pollen type, principally due to nectar secretion of *Citrus* sp. flowers, often before the maturity of stamens. *Citrus* pollen varied between 0.2% and 16.1%, with an average of 6.1%. The second group (group II: 23 samples, 19–41) was characterized by the absence of a *Citrus* sp./*A. sinensis* association and the significant presence of *A. ramosus* **T22** associated with *Pistacia lentiscus* **T20**, *Phillyrea* sp. **T24**, *Apiaceae* **T14** and *Brassicaceae* **T16**. *A. ramosus* displayed an extreme “under-represented” pollen type due to the flower form (nectar protected by a large base of long stamens that prevented contact with pollen during bee foraging) and the large pollen size. *Asphodelus* pollen was present in two samples of group I (0.5%–1.3%) and 16 samples of group II (0.2%–2.9%).

In the case of honey with the “under-represented” pollen type, the contribution of other nectariferous species could not be discounted. It had to note that some honeys samples possessed dominant nectariferous taxa (RF > 45%): *Trifolium* sp. **T9** for sample 2 and 3, *Echium* sp. **T13** for sample 4 and *Lotus* sp. **T7** for sample 38. The nectar contribution of these taxa could not be neglected. Otherwise, several taxa might take part in the honey composition for their high RF in the pollen spectrum: *Trifolium* sp. **T9** and *E. arborea* **T5** were characteristic for both groups (RF_max_ 53.5% and 35.5% for group I and 44.1% and 29.9% for group II, respectively); *Echium* sp. **T13**, *Prunus* form **T11** and *Viburnum tinus* **T26** possessed a higher RF_max_ in group I (71.1%, 24.1% and 16.2%, respectively) than in group II (30.1%, 3.3% and 3.7%, respectively), while *Lotus* sp. **T7**, *Genista* form **T6**, *Salix* sp. **T8**, *Lupinus angustifolius* **T41** and *Apiaceae* **T14** were higher in group II (FR_max_: 52.8%, 31.5%, 29.9%, 18.9% and 17.5%, respectively) than in group I (FR_max_: 8.7%, 11.8%, 12.9%, 3.5% and 4.5%, respectively).

A quantitative analysis showed that 32 samples possessed a pollen density between 20 and 100 × 10^3^ PG/10 g, eight samples were between 100 and 300 × 10^3^ PG/10 g and one sample (23) could be distinguished by high pollen density (600 × 10^3^ PG/10 g). Compared with the previous studies of Corsican “chestnut grove” and “*Erica arborea* spring maquis” honey (636.6 × 10^3^ PG/10 g and 177 × 10^3^ PG/10 g, respectively), the “spring” honey displayed a lower pollen density (90 × 10^3^ PG/10 g) [24,29]. Excluding sample 23, the average pollen density of “clementine” honeys (68 × 10^3^ PG/10 g) was slightly lower than that of other Corsican “spring” honeys (84 × 10^3^ PG/10 g) (Table 2). The decreasing pollen richness was in accordance with the pollen representation type in the spectrum of the predominant nectariferous taxa: “over-represented” (*C. sativa*), “normal” (*E. arborea*) and “under-represented” (*Citrus* sp.) types.

**Table 2:** Melissopalynological and physico-chemical characteristics of Corsican “clementine” honeys and other “spring” honeys.

Melissopalynological Data			Group I—“Clementine” Honeys 18 Samples (1–18)						Group II—Other “Spring” Honeys 23 Samples (19–41)					
			RF ^c^						RF ^c^					
No. ^a^	Type ^b^	Taxa	PR	Mean	Min.	Max.	SD	CV	PR	Mean	Min.	Max.	SD	CV
*Main nectariferous taxa*														
T18	N, P	*Citrus* sp.	100	6.1	0.2	16.1	5.2	86.6	-	-	-	-	-	-
T5	N, P	*Erica arborea*	89	7.5	0.3	35.5	10.2	135.4	83	8.0	0.2	29.7	7.6	94.9
T8	N, P	*Salix* sp.	78	5.8	0.6	12.9	4.5	77.7	70	6.7	0.2	29.9	9.3	139.4
T11	N, P	*Prunus* form	78	4.8	0.2	24.1	6.1	128.4	57	1.2	0.3	3.3	0.9	75.5
T7	N, P	*Lotus* sp.	72	2.7	0.4	8.7	2.5	91.9	78	7.2	0.3	52.8	12.1	167.5
T10	N, P	*Rubus* sp.	67	2.3	0.4	6.5	1.8	77.9	74	4.5	0.4	11.7	4.0	88.4
T9	N, P	*Trifolium* sp.	67	12.9	0.5	53.5	19.4	150.7	74	15.2	0.4	44.1	15.2	100.2
T6	N, P	*Genista* form	67	3.1	0.3	11.8	3.4	107.1	96	7.5	0.6	31.5	9.4	125.3
T13	N, P	*Echium* sp.	44	19.3	1.3	71.1	23.3	121.0	70	6.1	0.6	30.1	7.5	123.2
T26	N, P	*Viburnum tinus*	22	4.5	0.3	16.2	7.8	174.5	43	0.8	0.3	3.7	1.1	127.7
T14	N, P	*Apiaceae*	28	2.2	0.2	4.5	2.0	88.8	83	4.5	0.3	17.5	4.8	106.0
T22	N, P	*Asphodelus ramosus* subsp. *ramosus*	11	0.9	0.5	1.3	0.5	57.3	70	0.7	0.2	2.9	0.7	104.1
T16	N, P	*Brassicaceae* others	33	1.2	0.3	2.7	1.0	77.9	61	3.5	0.4	14.7	3.8	108.2
T17	N, P	*Lavandula stoechas*	39	1.7	0.5	4.4	1.4	81.9	57	1.8	0.4	10.1	2.6	142.4
T19	N, P	*Vicia* form	28	2.3	0.7	6.0	2.3	102.4	57	3.3	0.3	11.8	3.6	107.6
T41	N, P	*Lupinus angustifolius*	17	1.5	0.3	3.5	1.8	119.1	9	9.8	0.7	18.9	12.9	131.6
		Other nectariferous taxa	100	3.3	0.3	11.0	3.0	89.9	100	5.2	1.0	10.0	2.8	53.5
*Main only-polleniferous taxa*														
T15	P	*Actinidia sinensis*	100	4.6	0.3	16.1	4.6	99.7	9	0.5	0.3	0.7	0.3	55.3
T2	P	*Cistus* sp.	100	6.9	0.3	20.4	5.2	76.0	100	9.8	0.3	33.3	6.9	70.9
T1	P	*Quercus* sp.	100	16.5	0.8	35.7	10.7	64.9	100	10.6	1.3	29.0	8.1	77.2
T3	P	*Castanea sativa*	89	7.9	0.3	25.0	7.3	91.7	91	12.1	0.3	33.8	9.4	77.4
T4	P	*Fraxinus ornus*	89	6.1	0.5	22.3	6.6	108.4	91	1.1	0.3	3.3	1.0	87.6
T12	P	*Eucalyptus* sp.	72	3.3	0.4	15.5	4.1	125.9	57	1.0	0.3	3.4	1.0	99.3
T25	P	*Olea* sp.	50	0.8	0.4	1.3	0.3	38.9	26	1.3	0.6	3.6	1.1	84.3
T20	P	*Pistacia lentiscus*	11	0.6	0.6	0.6	0.0	8.2	70	3.3	0.5	9.3	2.7	80.4
T24	P	*Phillyrea* sp.	17	4.7	0.3	13.3	7.4	157.4	52	2.6	0.4	9.9	2.7	103.5
		Other only-polleniferous taxa	100	1.9	0.3	8.9	2.1	110.0	78	1.7	0.3	8.5	2.0	117.8
		Pollen density (10^3^ PG/10 g) ^d^		68	20	202	52	77		107	22	603	126	118
**Physico-chemical data ^e^**														
		Color		26.4	11.0	55.0	13.6	51.5		33.3	18.0	71.0	16.3	48.7
		Electrical conductivity		0.25	0.15	0.42	0.07	27.72		0.24	0.13	0.45	0.09	36.96

^a^ Taxa number is given in Table 1. ^b^ Type of taxa: P, polleniferous taxa; N, nectariferous taxa [5]. ^c^ Mean, Min., Max. values expressed as relative frequency RF (number of specify pollen counted/total pollen counted). ^d^ Pollen density expressed as the absolute number of pollen grains in 10 g of honey (10^3^ PG/10 g). ^e^ Unity of parameters: colour (mm Pfund); electrical conductivity (mS/cm).

### 3.2. Physicochemical Characteristics of Corsican “Spring” Honeys

Corsican “spring” honeys possessed light to very light colors. The mean value of coloration was 30.0 ± 15.4 mm Pfund, with great variation between 11.0 and 71.0 mm Pfund (Table 2). The two groups exhibited quite similar coloration values: 26.4 ± 13.6 mm Pfund for “clementine” honeys and 33.3 ± 16.3 mm Pfund for the other “spring” honeys. For each group, nine samples possessed a very light coloration value (<20.0 mm Pfund). Only one sample (17) of “clementine” honeys had a coloration value >50.0 mm Pfund while five samples (23, 35, 38, 39 and 41) of other “spring” honeys possessed coloration values between 50.0 and 71.0 mm Pfund.

The average electrical conductivity value of the honey samples was 0.25 ± 0.08 mS/cm with a variation of 0.13–0.45 mS/cm (Table 2). The electrical conductivity of the two groups was also quite similar: 0.25 ± 0.07 mS/cm for “clementine” honeys (range: 0.15–0.42 mS/cm) and 0.24 ± 0.09 mS/cm for other “spring” honeys (range: 0.13–0.45 mS/cm). Only three samples (17, 34 and 41) of these honeys had medium electrical conductivity (>0.4 mS/cm).

The coloration and electrical conductivity values of Corsican “spring” honeys were lower than those of “chestnut grove” and “*Erica arborea* spring maquis” honey ranges [5,24,29].

### 3.3. Chemical Variability of Corsican “Spring” Honeys

GC and GC/MS analysis of the headspaces of Corsican “spring” honeys allowed the identification of 43 compounds that accounted for 71.5%–96.8% of the total volatile composition (Table 3, Table S2-supplementary materials). It should be noted that the volatile fraction of “spring” honeys is rich in aldehyde (22.1%–63.1%) and alcohol (2.8%–40.2%) components.

**Figure 1:**
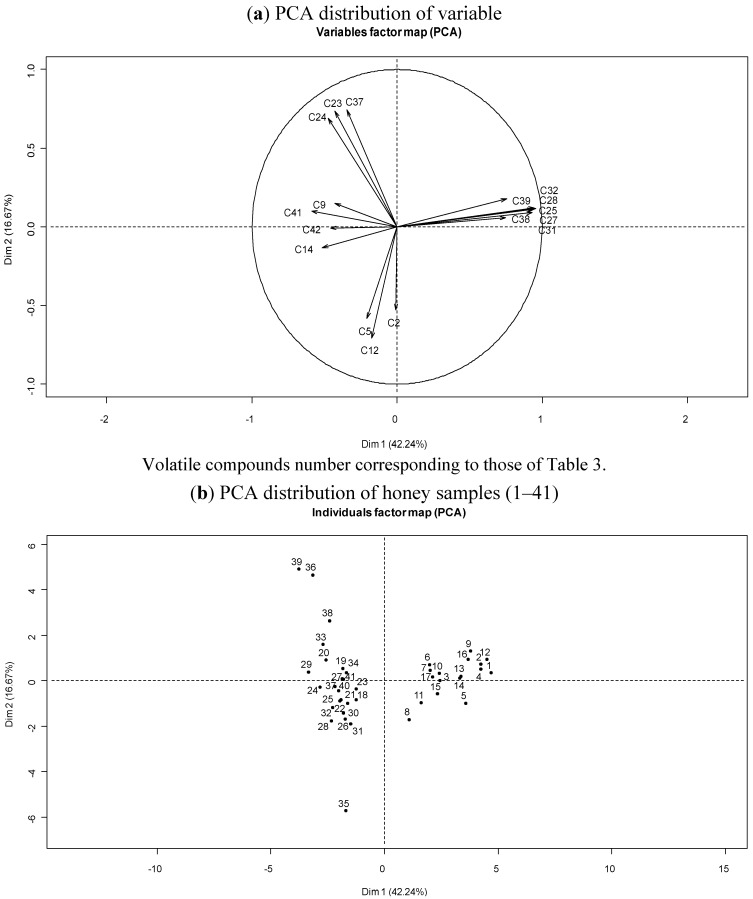
Principal component analysis (PCA) of Corsican “spring” honey volatile data.

To synthesize the chemical data, PCA was used to examine the relative distribution of the matrix of “spring” honey samples according to their volatile chemical compositions. The analyses included 17 compounds: two hydrocarbons (**C2** and **C12**), eight aldehydes (**C5**, **C9**, **C14**, **C25**, **C27**, **C28**, **C31** and **C32**), two ketones (**C23** and **C24**), two esters (**C38** and **C41**), two oxides (**C39** and **C42**) and one alcohol (**C37**). As shown in Figure 1a, the principal factorial plane (axes 1 and 2) accounted for 58.91% of the entire variability of the honey samples. Dimension 1 (42.24%) correlated negatively **C39** and negatively with other compounds. Dimension 2 (16.67%) correlated negatively with two hydrocarbons (**C2** and **C12**), two aldehydes (**C5** and **C14**) and one oxide **C42** and positively with with two ketones (**C23** and **C24**), three aldehydes (**C25**, **C27** and **C28**), one ester **C38** and one oxide other compounds.

The plot established according to the first two principal components suggested the existence of two main groups (Figure 1b). Group I contained 17 samples (1–17), which corresponded to the group of “clementine” honeys (except sample 18). This group I was characterized by the presence of lilac aldehyde isomers (**C25**, **C27** and **C28**), *p*-menth-1-en-9-al isomers (**C31** and **C32**) and methyl anthranilate **C38**, which were absent in the other honey samples (group II). It was rich in furan compounds (group I: 26.2% *versus* group II: 7.6%), but not in phenolic components (group I: 29.4% *versus* group II: 40.0%). Aldehyde components were also higher in group I (49.1%) than in group II (39.7%). Group II could be divided into subgroups IIa (five samples: 20, 33, 36, 38 and 39) and IIb (18 samples: 18, 19, 21–32, 34, 37, 40 and 41). These two subgroups were characterized by a greater abundance of phenolic compounds (group IIa: 39.9% and group IIb: 43.0%), but group IIa displayed a higher value for linear compounds (group IIa: 26.2% and group IIb: 19.2%). Additionally, subgroup IIa had a higher amount of aldehyde (group IIa: 40.7% and group IIb: 12.8%) and alcohol (group IIa: 35.6% and group IIb: 9.2%) compounds than subgroup IIb. This latter group displayed a greater abundance of ketones (group IIa: 4.3% and group IIb: 22.5%). Finally, sample 35 was characterized by 32.8% of hydrocarbons, whereas the abundance of hydrocarbons was not >25% in the other honey samples.

**Table 3:** Chemical composition of volatile fraction of Corsican “spring” honeys.

No ^a^	Components	RI ^b^	Group I “Clementine” Honeys ^c^			Group II “Not-Clementine” Honeys ^c^						Sample 35
						IIa			IIb			
			Mean ± SD ^d^	Min.	Max.	Mean ± SD ^d^	Min.	Max.	Mean ± SD ^d^	Min.	Max.	
**C1**	3-Methyl-3-buten-1-ol	704	2.9 ± 2.76	0.3	11.3	1.8 ± 1.19	0.7	3.7	1.8 ± 1.44	0.4	6.0	2.8
**C2**	Methyl-benzene	741	6.5 ± 4.45	1.5	15.6	4.1 ± 2.08	2.4	7.1	6.2 ± 4.08	1.5	17.3	10.4
**C3**	Hexanal	773	1.1 ± 0.46	0.5	2.0	1.6 ± 2.62	0.1	6.3	1.6 ± 1.13	0.3	4.5	1.9
**C4**	Octane	790	1.4 ± 1.08	0.3	4.7	0.9 ± 0.77	0.3	2.2	2.6 ± 1.63	0.7	5.6	1.4
**C5**	3-Furaldehyde	800	2.8 ± 1.56	0.6	5.9	3.5 ± 1.37	2.5	5.9	3.5 ± 1.78	1.9	8.3	18.5
**C6**	2-Methyl butanoic acid	858	0.8 ± 0.91	0.1	3.3	4.6 ± 3.21	1.1	7.6	2.9 ± 4.59	0.1	20.4	2.4
**C7**	2-Methyl octane	873	0.4 ± 0.28	0.1	0.9	0.5 ± 0.44	0.1	1.2	0.7 ± 0.38	0.1	1.7	1.6
**C8**	Nonane	893	0.7 ± 0.65	0.2	2.5	1.2 ± 0.35	0.8	1.7	1.5 ± 0.92	0.2	3.5	3.8
**C9**	Benzaldehyde	924	5.5 ± 3.56	2.4	17.9	10.4 ± 3.85	5.4	14.8	8.8 ± 4.89	2.5	18.4	3.0
**C10**	Hexanoic acid	969	0.7 ± 0.25	0.4	1.4	1.7 ± 1.68	0.3	3.9	1.2 ± 0.75	0.4	3.3	-
**C11**	Octanal	982	1.0 ± 0.47	0.2	2.1	0.6 ± 0.12	0.5	0.7	1.6 ± 1.45	0.5	6.6	1.0
**C12**	2,2,4,6,6-Pentamethylheptane	992	1.1 ± 0.60	0.4	2.4	0.6 ± 0.53	0.1	1.3	2.3 ± 1.83	0.2	5.2	15.6
**C13**	*p*-Methylanisol	995	0.9 ± 1.13	0.1	4.9	1.1 ± 1.10	0.3	3.0	0.9 ± 1.48	0.1	6.1	-
**C14**	Phenylacetaldehyde	1006	10.1 ± 10.68	0.8	39.1	16.5 ± 6.93	7.2	25.7	21.2 ± 7.83	3.7	36.2	13.0
**C15**	*p*-Cymene	1008	0.7 ± 0.29	0.1	1.0	0.9 ± 0.42	0.6	1.2	-	-	-	-
**C16**	Acetophenone	1037	0.2 ± 0.08	0.1	0.4	0.4 ± 0.21	0.2	0.5	0.3 ± 0.10	0.2	0.4	-
**C17**	*trans*-Furanoid-linaloxide	1049	1.5 ± 1.00	0.8	4.0	1.3 ± 1.25	0.5	3.5	2.4 ± 1.20	1.0	6.3	-
**C18**	*cis*-Furanoid-linaloxide	1064	1.1 ± 0.40	0.7	2.0	1.0 ± 0.29	0.7	1.4	1.1 ± 0.26	0.5	1.6	-
**C19**	β-Phenylethanol	1077	4.2 ± 1.54	2.2	5.8	1.6 ± 0.00	1.6	1.6	3.3 ± 1.67	2.1	5.8	-
**C20**	Nonanal	1079	2.7 ± 1.73	0.9	7.6	1.8 ± 1.28	0.4	3.5	3.0 ± 2.29	0.5	7.2	2.9
**C21**	Linalol	1084	2.4 ± 1.82	0.2	6.6	1.3 ± 1.62	0.3	3.2	12.5 ± 10.42	2.1	32.3	tr
**C22**	Hotrienol	1085	4.1 ± 4.39	0.7	10.5	-	-	-	9.7 ± 0.00	9.7	9.7	-
**C23**	Isophorone	1087	2.8 ± 1.54	0.2	4.9	18.2 ± 8.02	8.8	29.3	3.3 ± 3.5	0.1	9.6	-
**C24**	4-Oxoisophorone	1102	0.9 ± 0.33	0.3	1.4	4.2 ± 1.87	2.3	6.4	1.5 ± 0.99	0.3	5.0	-
**C25**	(2*S*,2′*S*,5′*S*)-Lilac aldehyde	1112	5.4 ± 2.36	1.3	8.9	-	-	-	1.5 ± 0.00	1.5	1.5	-
**C26**	Dihydrolinalool	1116	1.2 ± 0.66	0.5	3.0	-	-	-	1.1 ± 0.00	1.1	1.1	-
**C27**	(2*R*,2′*S*,5′*S*)-Lilac aldehyde	1121	10.5 ± 3.66	4.9	16.5	-	-	-	2.4 ± 0.00	2.4	2.4	-
**C28**	(2*R*,2′*R*,5′*S*)-Lilac aldehyde	1134	4.8 ± 1.85	2.2	8.1	-	-	-	1.1 ± 0.00	1.1	1.1	-
**C29**	Octanoic acid	1167	1.7 ± 1.55	0.3	6.0	0.9 ± 0.38	0.3	1.3	1.6 ± 0.78	0.7	3.8	4.7
**C30**	Decanal	1174	1.2 ± 0.67	0.2	2.8	0.6 ± 0.25	0.2	0.8	1.5 ± 0.54	0.6	2.3	-
**C31**	*p*-Menth-1-en-9-al (isomer 1)	1184	1.9 ± 0.41	1.2	2.7	-	-	-	-	-	-	-
**C32**	*p*-Menth-1-en-9-al (isomer 2)	1186	1.7 ± 0.46	0.5	2.5	-	-	-	-	-	-	-
**C33**	*p*-Anisaldehyde	1208	0.7 ± 1.19	0.1	4.6	0.9 ± 0.37	0.3	1.1	0.4 ± 0.21	0.2	0.8	-
**C34**	2,3,5-Trimethylphenol	1248	0.4 ± 0.30	0.1	1.1	1.0 ± 0.69	0.4	2.0	0.8 ± 0.68	0.1	2.0	-
**C35**	4-*n*-Propylanisol	1264	1.6 ± 1.78	0.2	5.7	2.4 ± 1.29	0.8	4.3	3.8 ± 2.45	1.4	6.3	-
**C36**	Nonanoic acid	1271	2.7 ± 1.39	0.5	4.9	2.6 ± 0.94	1.4	3.7	3.1 ± 1.24	1.3	6.4	-
**C37**	3,4,5-Trimethylphenol	1290	0.5 ± 0.32	0.2	1.4	5.4 ± 2.71	2.9	9.4	0.5 ± 0.67	0.1	2.0	-
**C38**	Methyl anthranilate	1300	1.4 ± 0.96	0.2	3.5	-	-	-	-	-	-	-
**C39**	*cis-p*-Mentha-1(7),8-dien-1-hydroperoxide	1348	0.4 ± 0.14	0.2	0.7	-	-	-	-	-	-	-
**C40**	Decanoic acid	1362	1.2 ± 0.45	0.6	2.1	1.3 ± 0.75	0.1	1.9	1.7 ± 1.50	0.6	6.8	3.8
**C41**	Methyl 3,5-dimethoxybenzoate	1494	-	-	-	0.4 ± 0.26	0.2	0.7	0.5 ± 0.19	0.3	0.8	-
**C42**	Methyl syringate	1722	-	-	-	0.5 ± 0.50	0.1	1.4	0.9 ± 1.17	0.1	4.1	-
**C43**	Tricosane	2305	0.3 ± 0.17	0.1	0.5	0.5 ± 0.00	0.5	0.5	0.5 ± 0.22	0.2	0.7	-
	*Total identification (%)*		84.2 ± 6.95	71.5	94.5	91.2 ± 5.43	84.2	96.8	86.3 ± 5.49	78.8	96.7	86.8
	*Total peak area(10^6^) ^e^*		3.8 ± 1.96	1.3	7.4	2.9 ± 1.27	1.6	4.5	2.4 ± 1.08	0.8	4.4	0.3
	Hydrocarbons		10.6 ± 5.23	4.7	20.8	7.7 ± 3.59	4.8	13.5	13.3 ± 5.88	5.0	23.9	32.8
	Oxygenated compounds		73.6 ± 7.23	58.2	82.8	83.6 ± 4.11	79.2	90.3	73.7 ± 7.56	58.1	81.7	54.0
	Phenolic compounds		29.4 ± 12.3	12.6	59.3	43.0 ± 7.39	34.8	53.0	39.9 ± 11.22	23.2	60.4	26.4
	Furan compounds		26.2 ± 7.85	12.2	38.6	5.8 ± 2.83	3.9	10.8	7.5 ± 1.99	4.3	11.0	18.5
	Linear compounds		21.0 ± 7.08	11.3	36.6	19.2 ± 3.88	14.8	23.4	26.2 ± 9.63	11.3	53.4	41.9
	Terpenic compounds		31.0 ± 9.88	15.4	52.2	3.5 ± 2.64	1.7	8.1	13.6 ± 13.05	1.5	45.8	0
	Ketones		2.5 ± 2.14	0	6.0	22.5 ± 9.67	11.6	35.7	4.3 ± 3.77	0.8	11.6	0
	Aldehydes		49.1 ± 8.03	34.4	63.1	35.6 ± 9.73	26.1	47.7	40.7 ± 9.78	22.1	52.3	40.3
	Esters		1.4 ± 0.96	0.2	3.5	0.2 ± 0.29	0	0.7	0.3 ± 0.27	0	0.8	0
	Alcohols		9.7 ± 6.17	3.3	27.8	9.2 ± 3.3	4.9	12.7	12.8 ± 10.40	3.0	40.2	2.8
	Acids		6.8 ± 3.31	0.4	15.4	9.7 ± 5.23	3.0	15.1	10.2 ± 5.28	5.6	27.0	10.9
	Oxides		5.2 ± 2.76	2.5	12.6	6.3 ± 2.45	3.0	9.3	5.5 ± 2.64	3.3	14.3	0

^a^ Order of elution is given on apolar coloumn (Rtx-1). ^b^ Retention indice on the Rtx-1 apolar column. ^c^ Group number was given in “Chemical variability of Corsican “spring” honeys”. ^d^ Means ± SD, Min. and Max. values expressed as percentages. ^e^ Total peak area was expressed in arbitrary units.

### 3.4. Botanical Origin and Volatile Composition of Corsican “Spring” Honeys

The 17 samples of “clementine” honeys (group I: 1–17) could be distinguished from other “spring” honeys (group II) by the presence of three lilac aldehydes (**C25**, **C27** and **C28**) and two *p*-menth-1-en-9-al isomers (**C31** and **C32**) (Table 3, Table S2-supplementary materials). These honey samples were dominated by phenolic compounds (12.6%–59.3%), followed by furan compounds (12.2%–38.6%) and linear compounds (11.3%–36.6%). The main components were phenylacetaldehyde **C14** (0.8%–39.1%), methyl-benzene **C2** (1.5%–15.6%), (2*R*,2′*S*,5′*S*)-lilac aldehyde **C27** (4.9%–16.5%), (2*S*,2′*S*,5′*S*)-lilac aldehyde **C25** (1.3%–8.9%), benzaldehyde **C9** (2.4%–17.9%) and (2*R*,2′*R*,5′*S*)-lilac aldehyde **C28** (2.2%–8.1%). Low amounts of methyl anthranilate **C38** (0.2%–3.5%) were found in the volatile fraction of the “clementine” honeys analyzed. This component is a known chemical marker of *Citrus* (species not specified) unifloral honey [13]. Additionally, various linalool derivatives, such as linalool oxides, lilac aldehydes and/or *p*-menth-1-en-9-al isomers, have also been reported as characteristic compounds of citrus unifloral honeys from Spain and Greece [15,16,30,31,32,33]. These compounds were also identified in the volatile components of Corsican “clementine” honeys. Conversely, some other linalool derivatives, such as lilac alcohol isomers (previously reported in the Spanish and Greek citrus honeys), were not detected in our honey samples. Alissandrakis *et al*. [15] showed that methyl anthranilate and lilac aldehydes could be found in honeys of mixed botanical origin with the presence of citrus nectar. These volatile compounds were also detected in the honey samples 2–4 in which were found the RF_max_ of *Trifolium* sp. **T9** and *Echium* sp. **T13** taxa. Chemical investigation showed that these honey samples displayed the similar volatile composition of “clementine” honey. As these two taxa could provide great quantity of nectar and pollen [34], it appeared that they played only a polleniferous role in these honey samples.

The volatile composition of *C. sinensis* × *reticulata* flowers has not been reported previously. The HS-SPME fraction of clementine flowers is characterized by 29 compounds, which accounted for 75.5%–87.0% of the volatile composition (Table 4). Linalool (9.6%–22.6%), sabinene (13.4%–19.6%), dihydrolinalool (8.5%–14.8%) and myrcene (5.6%–6.5%) were identified as the main compounds. Linalool and dihydrolinalool were also found in low concentrations in the volatile fraction from “spring clementine” honey samples. Methyl anthranilate was detected in the volatile fraction of Corsican clementine flowers (0.1%–0.3%) and corresponding honeys.

The decrease in linalool amount and the occurrence of other linalool derivates (hotrienol, linalool oxides, lilac aldehyde isomers and *p*-menth-1-en-9-al isomers) in honey samples could be explained by the enzymatic degradation of linalool by some pathways [15]: (1) linalool can be transformed to 8-hydroxylinalool isomers by enzymatic hydroxylation at the C8 position, and then hotrienol; (2) 8-hydroxylinalool can be transformed to lilac aldehyde via (*E*)-8-oxolinalool and lilac alcohols, or *p*-menth-1-en-9-al via 8-hydroxygeraniol and (3) linalool can also be transformed via 6,7-hydroxylinalool into furanoid linalool oxide isomers under acidic conditions or by heating. These results were in accordance with those previously reported on the volatile fraction of citrus flowers and corresponding honeys [16]. It demonstrated that the flowers from *Citrus* species (orange, tangerine and sour orange) had high amounts of linalool (51.6%–80.6%) and that the honeys consisted of more than 80% of linalool derivatives (lilac aldehydes and lilac alcohols).

**Table 4:** Chemical composition of volatile fraction of clementine and asphodel flowers.

Components ^a^	RI(Lit) ^b^	RI ^c^	Clementine Flower ^d^			Asphodel Flower ^e^			Identification ^g^
			Mean ± SD ^f^	Min.	Max.	Mean ± SD ^f^	Min.	Max.	
3-Furaldehyde	799	800	-	-	-	1.0 ± 0.87	0.5	2.7	RI, MS
Furfural	831	836	-	-	-	3.5 ± 1.26	1.7	5.2	RI, MS
2-Furanmethanol	839	842	-	-	-	2.1 ± 1.48	0.8	4.7	RI, MS, Ref
Heptanal	882	876	-	-	-	5.4 ± 2.46	3.1	9.5	RI, MS
α-Thujene	924	922	1.2 ± 0.21	1.0	1.4	-	-	-	RI, MS
α-Pinene	932	931	3.6 ± 2.46	2.0	6.4	-	-	-	RI, MS
Benzaldehyde	929	933	-	-	-	2.7 ± 0.78	1.4	3.7	RI, MS
Tetrahydro-citronellene	937	935	6.8 ± 4.90	3.3	12.4	-	-	-	RI, MS, Ref
β-Citronellene	943	940	2.2 ± 0.15	2.0	2.3	-	-	-	RI, MS
Octen-3-ol	962	955	-	-	-	0.2 ± 0.05	0.1	0.2	RI, MS
Furfuryl acetate	964	959	-	-	-	0.7 ± 0.31	0.5	1.3	RI, MS, Ref
Sabinene	973	958	16.8 ± 3.14	13.4	19.6	-	-	-	RI, MS
2-Pentylfuran	973	966	-	-	-	0.8 ± 1.00	0.2	2.8	RI, MS
β-Pinene	978	972	1.5 ± 1.36	0.4	3.0	-	-	-	RI, MS
Myrcene	987	979	6.1 ± 0.45	5.6	6.5	-	-	-	RI, MS
Octanal	981	980	-	-	-	7.0 ± 3.12	3.5	12.6	RI, MS
(*Z*)-3-Hexenyl acetate	989	984	-	-	-	21.6 ± 14.27	5.2	41.8	RI, MS
(*E*)-3-Hexenyl acetate	1002	994	-	-	-	0.8 ± 0.54	0.1	1.5	RI, MS
α-Phellandrene	1002	995	1.5 ± 0.23	1.4	1.8	0.3 ± 0.12	0.1	0.4	RI, MS
α-Terpinene	1013	1008	0.6 ± 0.44	0.3	1.1	-	-	-	RI, MS
Phenylacetaldehyde	1012	1009	-	-	-	0.9 ± 0.67	0.2	2.1	RI, MS
*p*-Cymene	1015	1011	0.6 ± 0.10	0.5	0.7	-	-	-	RI, MS
*p*-Menth-1-ene	1017	1018	0.5 ± 0.15	0.4	0.7	-	-	-	RI, MS
Limonene	1025	1020	1.5 ± 0.70	0.8	2.2	-	-	-	RI, MS
(*Z*)-β-Ocimene	1029	1024	0.1 ± 0.06	0.1	0.2	-	-	-	RI, MS
(*E*)-2-Octenal	1034	1034	-	-	-	0.4 ± 0.29	0.1	0.8	RI, MS
(*E*)-β-Ocimene	1041	1036	2.6 ± 2.21	0.9	5.1	-	-	-	RI, MS
γ-Terpinene	1051	1047	1.1 ± 0.51	0.5	1.5	-	-	-	RI, MS
*trans*-Sabinene hydrate	1053	1050	1.0 ± 0.36	0.7	1.4	-	-	-	RI, MS
1-Octanol	1063	1057	-	-	-	6.0 ± 1.93	2.8	8.8	RI, MS
Terpinolene	1082	1078	0.1 ± 0.06	0.1	0.2	-	-	-	RI, MS
Nonanal	1076	1081	-	-	-	25.8 ± 10.1	16.5	38.2	RI, MS
Linalool	1086	1086	17.8 ± 7.14	9.6	22.6	1.7 ± 0.21	1.5	1.8	RI, MS
Tetrahydrolinalool	1099	1095	4.1 ± 3.07	0.7	6.7	-	-	-	RI, MS, Ref
Dihydrolinalool	1118	1114	10.8 ± 3.50	8.5	14.8	-	-	-	RI, MS, Ref
(*E*)-2-Nonen-1-ol	1149	1153	-	-	-	2.2 ± 1.65	0.6	4.6	RI, MS
1-Phenylethyl acetate	1166	1163	-	-	-	0.1 ± 0.05	0.1	0.2	RI, MS
Terpinen-4-ol	1164	1164	0.3 ± 0.20	0.1	0.5	-	-	-	RI, MS
α-Terpineol	1176	1173	tr	tr	tr	-	-	-	RI, MS
Decanal	1180	1182	-	-	-	1.6 ± 0.74	0.7	2.5	RI, MS
Undecanal	1285	1285	-	-	-	1.0 ± 1.03	0.2	2.8	RI, MS
Methyl anthranilate	1308	1302	0.2 ± 0.10	0.1	0.3	-	-	-	RI, MS
(*E*)-Jasmone	1356	1360	tr	tr	tr	-	-	-	RI, MS
Isocaryophyllene	1409	1405	tr	tr	tr	-	-	-	RI, MS
(*E*)-β-Farnesene	1446	1442	tr	tr	tr	-	-	-	RI, MS
(*E*,*E*)-α-Farnesene	1498	1492	0.1 ± 0.00	0.1	0.1	-	-	-	RI, MS
(*E*)-Nerolidol	1553	1548	tr	tr	tr	-	-	-	RI, MS
Heptadecane	1700	1698	0.2 ± 0.10	0.1	0.3	-	-	-	RI, MS
**Total identification (%)**			81.2 ± 5.75	75.5	87.0	85.9 ± 2.66	82.4	90.1	
Hydrocarbons			48.0 ± 8.16	39.7	56.0	-	-	-	
Oxygenated compounds			33.1 ± 11.88	19.5	41.3	85.9 ± 2.66	82.4	90.1	
Phenolic compounds			0.2 ± 0.1	0.1	0.3	3.7 ± 0.99	1.8	4.5	
Furan compounds			-	-	-	8.2 ± 4.42	4.3	16.7	
Linear compounds			0.2 ± 0.1	0.1	0.3	73.9 ± 5.41	65.0	79.3	
Terpenic compounds			80.8 ± 5.75	75.1	86.6	0.8 ± 0.85	0.1	1.9	
Ketones			tr	-	tr	-	-	-	
Aldehydes			-	-	-	43.8 ± 13.47	31.2	62.8	
Esters			0.2 ± 0.1	0.1	0.3	23.2 ± 14.58	5.8	43.1	
Alcohols			32.9 ± 11.8	19.4	41.1	11 ± 4.13	4.3	17	

^a^ Order of elution is given on apolar coloumn (Rtx-1). ^b^ Retention indice of literature on the apolar column reported from references [27,28]. ^c^ Retention indice on the Rtx-1 apolar column. ^d^ Six clementine flower specimens were collected from Corsica oriental plain. ^e^ Six Asphodele flower specimens were collected from six localities of Corsica. ^f^ Means ± SD, Min. and Max. values expressed as percentages; tr trace (< 0.05%), ^g^ RI, Retention indice; MS, mass spectra in electronic impact mode. Ref., compounds identified from commercial data libraries: Konig *et al.* [27] (Samples 8, 34 and 35) and NIST [28] (Samples 3 and 11).

The 23 “not-clementine” honey samples (group II) were dominated by phenolic compounds (23.2%–60.4%) followed by linear compounds (11.3%–53.4%). The main compounds were phenylacetaldehyde **C14** (3.7%–36.2%), benzaldehyde **C9** (2.5%–18.4%) and methyl-benzene **C2** (1.5%–17.3%). Furanic compounds (average: 7.5%) were less abundant than in “clementine” honeys (average: 26.2%), and acid components (average: 10.3%) were more abundant than in the “clementine” honeys (average: 6.8%). To our knowledge, only one previous report focused on the volatile fraction of asphodel unifloral honeys from Sardinia [18]. Methyl syringate was detected in asphodel nectar in high concentrations and was therefore considered a marker of asphodel honeys [19]. A low content of this component (**C42**: 0.1%–4.1%) was reported in the volatile fraction of “spring” honey samples (18–21, 24–30, 32, 33 and 36–41). Additionally, the amount of methyl syringate was unrelated to the presence of *Asphodelus* pollen in the pollen spectrum. This result could be explained by the extreme “under-represented” type of *Asphodelus* pollen in Corsican “spring” honeys and/or by other nectar contributions in these honeys. The sample 18 exhibited the association of *Citrus* sp. and *A. sinensis*; it was grouped with the “not-clementine” honey. In this sample, the citrus nectar contribution was less important than in “clementine” honeys in accordance with the lower concentrations of lilac aldehyde isomers.

To our knowledge, the volatile composition of *A. ramosus* subsp. *ramosus* flowers is reported here for the first time (Table 4). The HS-SPME volatile fraction of asphodel flowers was dominated by oxygenated compounds, especially linear compounds. Nonanal (16.5%–38.2%), (*Z*)-3-hexenyl acetate(5.2%–41.8%), octanal (3.5%–12.6%), 1-octanol (5.7%–8.8%) and heptanal (3.1%–9.5%) were identified as major compounds. The two main components of the honey volatile fraction (phenylacetaldehyde and benzaldehyde) were detected in low concentrations in the flowers. Moreover, methyl syringate (a marker of asphodel honey) was not detected in the flowers analyzed. This result showed that a direct relationship between the volatile fractions of asphodel flowers and the corresponding “spring” honeys could not be established using HS-SPME analysis.

Finally, the characteristic compounds of the volatile fraction of Corsican “chestnut grove” (acetophenone and 2-aminoacetophenone) [24] and “*Erica arborea* spring maquis” (*p*-anisaldehyde and 4-propylanisol) honeys [29] were found in low concentrations or not detected in the “spring” honeys studied.

### 3.5. Correlation of Melissopalynological and Chemical Data

To identify relationships between the melissopalynological analysis and volatile composition data of honey samples, CCA was applied on the matrix linked the relative amounts of the 17 volatile compounds (previously used in section “Chemical variability of Corsican “spring” honeys”) and the relative frequency (explanatory variables) of eight nectariferous taxa (**T7**–**T9**, **T11**, **T13**, **T14**, **T18** and **T22**).

**Figure 2:**
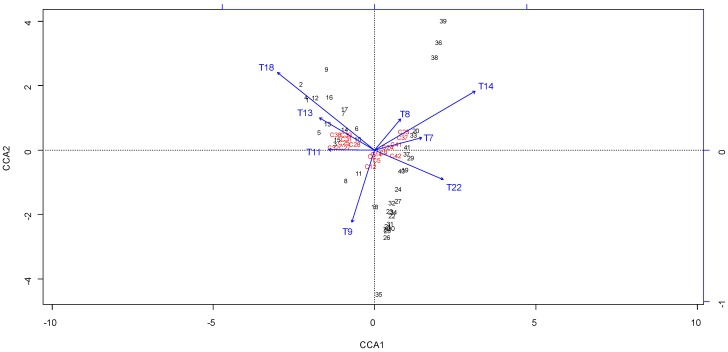
Correlation between melissopalynological and volatile data of “spring” honey by canonical correspondence analysis (CCA).

The correlations between the volatile composition and melissopalynolgical data were show in Figure 2. The first CCA axis was negatively related *Trifolium* sp. **T9**, *Prunus* form **T11**, *Echium* sp. **T13** and *Citrus* sp. **T18** to methyl-benzene **C2**, 2,2,4,6,6-pentametylheptane **C12**, three lilac aldehydes (**C25**, **C27** and **C28**), two *p*-menth-1-en-9-al isomers (**C31** and **C32**), methyl antranilate **C38** and *cis*-*p*-mentha-1(7),8-dien-1-hydroperoxide **C39**. The second axis negatively related *Trifolium* sp. **T9** and *Asphodelus* **T22** to methyl-benzene **C2**, 3-furaldehyde **C5**, Benzaldehyde **C9**, 2,2,4,6,6-pentametylheptane **C12**, phenlyacetaldehyde **C14** and methyl syringate **C42**.

The sample distribution showed the occurrence of two main groups, group I (17 samples: 1–17) and group II (24 samples: 18–41), which correspond to the groups defined in “Determination of geographical and botanical origins of Corsican “spring” honeys” Group I was characterized not only by the significant presence of lilac aldehyde isomers (**C25**, **C27** and **C28**), *p*-menth-1-en-9-al isomers (**C31** and **C32**) and methyl anthranilate **C38**, but also by the high abundance of taxa: *Citrus* sp. **T18**, *Echium* sp. **T13** and *Prunus* form **T11** (group I: 6.3%, 9.5% and 3.8% *versus* group II: 0.1%, 4.9% and 0.8%, respectively). According to the literature [15,16,30,31,32,33], all these compounds had been considered as characteristic components of citrus honey. From these results, it appeared that the other nectariferous taxa *Echium* sp. and *Prunus* form displayed a polleniferous role in these honey samples.

Group II included 24 samples that had great diversity. According to the sample distribution, we could distinguish 20 honey samples (18–34, 37, 40 and 41), which had higher values of phenylacetaldehyde **C14** and methyl syringate **C38** (21.4% and 0.7%, respectively). These honeys were also characterized by numerous herbaceous taxa with potential for nectar contribution, such as *Lotus* sp. **T7**, *Salix* sp. **T8**, *Apiaceae* **T14** and *Asphodelus* **T22** (3.5%, 4.4%, 3.2% and 0.6% *versus* I: 2.0%, 4.6%, 0.4% and 0.03%, respectively). As previously reported in literature data [19], the nectar contribution of *Asphodelus* **T22** in these honey samples was characterized by the presence of methyl syringate **C38**. In the same way, phenylacetaldehyde **C14** was reported as main volatile compound of *Salix* honeys [35] and *Asphodelus* honey [18]. For the other nectariferous species *Lotus* sp. **T7** and *Apiaceae* **T14**, no chemical markers of nectar contribution was reported in previous studies.

Variables: taxa number corresponding to those of Table 2 and Table 3; volatile components number corresponding to those of Table 3. In the CCA plot, location of each sample indicated its compositional similarity to each other; volatile components locations indicated the similarity of their distribution to each other; length of taxa indicated the importance to the ordination, and the direction of taxa vector indicated its correlation with each axes. The perpendiculars drawn from volatile components to taxa give approximate ranking of volatile components response to the taxa variables.

## 4. Conclusions

Corsican “spring” honeys can be classified into two categories according to melissopalynological analysis: (1) honeys characterized by the association of cultivated plants, especially *C. sinensis* × *reticulata* with other *Citrus* species, *A. sinensis* and other fruit trees; (2) honeys without cultivated taxa, but with herbaceous species (*A. ramosus* subsp. *ramosus*, *Trifolium* sp., *Echium* sp., *Apiaceae*, *Brassicaceae*, *Lotus* sp., *etc.*), low shrub species (*Rubus* sp. and *Lavandula stoechas*) and some polleniferous taxa with precocious flowering (*P. lentiscus* and *Phillyrea* sp.).

Analysis of the volatile fraction of “spring” honeys also demonstrated the existence of two main groups in this range. The volatile fractions were often characterized by high amounts of phenylacetaldehyde, benzaldehyde and methyl-benzene. However, the chemical composition of “clementine” honeys was dominated by three lilac aldehyde isomers that were absent in the “not-clementine” honeys. The statistical analysis showed clearly that the “clementine” honeys were characterized by high volatile content (total peak area), methyl anthranilate, lilac aldehydes, *p*-menth-1-en-9-al isomers and some cultivated taxa, while the “not-clementine” honeys were characterized by phenylacetaldehyde, methyl syringate and complex taxa associations. The richness of linalool derivatives in the volatile fraction of clementine flowers suggested biochemical transformation occurring during honeybee activity or honey conservation in the hive. 

Finally, it appeared that melissopalynological analysis was necessary for the certification of geographical origin and was useful for the determination of botanical origin. Moreover, analysis of the volatile composition could be used to specify the characteristics of volatile compounds in relation to the predominance and/or complexity of botanical origins of the product, especially when nectariferous species have an “under-represented” pollen type in the pollen spectrum, such as *Citrus* sp. or *Asphodelus* sp.

## Supplementary Materials

Supplementary File 1
